# Heavy metals, noradrenaline/adrenaline ratio, and microbiome-associated hormone precursor metabolites: biomarkers for social behaviour, ADHD symptoms, and executive function in children

**DOI:** 10.1038/s41598-025-00680-5

**Published:** 2025-05-30

**Authors:** Kristin Krajewski

**Affiliations:** Institute of Psychology, University of Education, Ludwigsburg, Germany

**Keywords:** Gut-brain axis, Neurotransmitter, Organic acids, Metabolome, Toxins, Self-regulation, Human behaviour, Diagnostic markers, Risk factors

## Abstract

The gut microbiome significantly influences physical and mental health, including the synthesis and metabolism of hormones and the detoxification of heavy metals, which are linked to behavioural disorders. This study investigated the associations of these biological factors with the behaviour of primary school children, specifically examining the effects of heavy metals, catecholamines, and microbiome-associated metabolites of dopamine, noradrenaline, adrenaline, and thyroxine precursors. Urine samples from 87 unselected primary school children were analysed to assess heavy metal load (arsenic, cadmium, lead, mercury), noradrenaline/adrenaline ratio, and microbiome-associated metabolites of phenylalanine, tyrosine and L-dopa (3-phenylpropionic acid, p-OH-phenylacetic acid, 4-hydroxybenzoic acid, 3,4-dihydroxyphenylpropionic acid). Three months later, executive functions, ADHD symptoms (inattention, hyperactivity and impulsivity), and social behaviour were evaluated via parent and teacher questionnaires. In a path model, heavy metal load, microbiome-associated metabolites, and the noradrenaline/adrenaline ratio measured in urine accounted for 32% of social behaviours. Microbiome-associated metabolites predicted 11% of the variance in executive functions and 17% in ADHD symptoms. Executive functions shared 55% of the variance with ADHD symptoms and 17% with social behaviours. Children with the lowest social behaviours had a sixfold increase in the odds of high heavy metal loads and a 3.4-fold increase in the odds of elevated microbiome-associated metabolites. Similarly, children with the most compromised executive functions had a threefold increase in the odds of such high metabolite levels. Overall, the results indicate that children’s social behaviours are influenced by heavy metal accumulation, catecholamine balance, and the microbiome-associated metabolism of amino acids, that are crucial for producing stress and thyroid hormones.

## Introduction

### Microbiome, hormones, health, and behaviour

In recent years, worldwide studies of the microbiome have demonstrated the substantial influence of the intestinal microbiota on both physical and mental health. The microbiome consists of all the genes and genomes of approximately 100 trillion microorganisms coexisting in the gut and is understood to be central to the immune system^1^. In particular, the beneficial microbiota forms a protective barrier that prevents toxins from entering the bloodstream through the intestine^2^. Moreover, among their various roles, they are responsible for the production of neurotransmitters (e.g., dopamine, noradrenaline, serotonin, GABA, acetylcholine, and histamine)^3,4^, B vitamins^5^, and omega-3 fatty acids^6^. These compounds are essential for ensuring optimal brain and nervous system health. Recent studies have underscored the critical role of omega-3 fatty acids in maintaining brain functionality, showing that a deficiency in these fats is increasingly linked to the development of neuropsychiatric disorders^7^. Furthermore, an imbalance in the intestinal microbiota is associated with immune system suppression, poorer sleep quality^8^, thyroid disorders^9^ and imbalances in catecholamines such as the noradrenaline/adrenaline ratio^10^. As such, microbiome changes are related to a range of chronic diseases, including chronic fatigue syndrome (CFS)^11^, attention deficit/hyperactivity disorder (ADHD)^12,13^, autism^14^, depression, anxiety, and schizophrenia^15^. In line with these findings, Rundek and colleagues reported that microbiome changes lead to systemic and neuroinflammation, subsequently resulting in cognitive decline^16^. Moreover, preliminary data indicate a positive correlation between microbiome changes and erratic social behaviour. Specifically, germ-free mice, which lack detectable gut microbiota, exhibit not only atypical brain function but also abnormal social interactions^17^. In studies involving healthy humans, the crucial gut microbiota *Bifidobacterium longum* 1714 has been shown to modulate social stress and memory^18^, further underscoring the microbiome’s impact on behaviour. Lastly, in human infants, a dysbalanced microbiota is associated with fear behaviour^19^, illustrating that these relationships may begin in early development. Interestingly, some bacterial species, such as *Klebsiella pneumoniae,* which are often labeled as ‘opportunistic pathogens’, may also function as ‘beneficial’ or detoxifying agents in the microbiome, as they can potentially biodegrade and metabolize microplastics in the human gut^20^.

### Catecholamines and microbially associated precursor metabolites as markers for brain function and behaviour

Given the complex interplay between the microbiome and the nervous system, it is essential not only to examine microbiota-related compounds but also to focus on specific biochemical markers that bridge microbial activity and brain function. Catecholamines, including noradrenaline (norepinephrine), adrenaline (epinephrine) and dopamine, are hormones that are involved in activating the sympathetic nervous system and play crucial roles in cognition and behaviour^21^. Imbalances in catecholamines and thyroid dysfunction have been linked to behavioural issues such as social attitudes, moral judgements^22^, ADHD and autism^23^. To investigate the role of catecholamines in these complex behavioural and physiological relationships, reliable measurements of their levels and activity are essential. The measurement of noradrenaline and adrenaline in urine, which has a long-standing history dating back to the 1950s^24^, provides valid indicators of sympathetic activity and is associated with physical and behavioural issues. For example, the urinary noradrenaline-to-adrenaline ratio is correlated with heart rate and thyroid function^25^, mental workload and physical training responses^26,27^, epilepsy^28^, and various psychiatric and gastrointestinal disorders^29^. As key players in sympathetic nervous system activity, catecholamines are closely linked to both cognitive functions and behavioural regulation. To fully understand their impact, it is also important to consider the metabolic precursors involved in their synthesis. Phenylalanine, tyrosine, and L-dopa not only serve as foundational substrates for catecholamine production but also reveal important links to neurological function and various metabolic disorders. The first two, which are amino acids, also act as precursors for the thyroid hormone thyroxine. Phenylketonuria, a disease resulting from a defect in the metabolism of these precursors, is associated not only with intellectual disabilities and hyperactive behaviours with possible autistic features^30^ but also with gut-microbiome alterations^31^. With the discovery of phenylketonuria in 1934, the focus has been directed towards an abnormal accumulation of metabolites in the urine^32^ in cases of such metabolic disorders. A recent study reported increased urinary levels of phenylalanine and decreased tyrosine levels, along with elevated concentrations of phenylalanine metabolites, even those linked to autism^33^. Another study highlighted that urinary metabolites of phenylalanine, tyrosine and L-dopa are also associated with the microbiome’s presence or activity, as they may either be directly affected by the microbiota or interact with it. High urinary levels of specific phenylalanine and tyrosine metabolites may indicate an overabundance^34^ of gut bacteria. As a result, urinary metabolites of phenylalanine, tyrosine, and L-dopa may act as markers of microbiome-associated activity in catecholamine pathways, with levels varying on the basis of a person’s genetics, diet, and other epigenetic and environmental influences^35^. However, these metabolites may also originate from dietary sources or other metabolic pathways^36^. Interestingly, they also have antimicrobial and anti-inflammatory properties, supporting the immune system’s defence against harmful pathogens^37–40^.

### Heavy metal load, hormones, and behaviour

In recent years, several behavioural disorders have been linked not only to an “unhealthy” gut microbiome or a modified catecholamine synthesis pathway but also to toxic heavy metal overload in the body. Arsenic, cadmium, lead, and mercury have been identified as particularly harmful. Autism has been associated with lead, arsenic, and mercury^14^; ADHD^41^, hostility, oppositional defiant behaviours, and poor emotional regulation have been linked to lead^42^. Social, attentional, and intellectual problems have been associated with cadmium^43^; and developmental disorders have been related to mercury^44^, lead, and arsenic^45,46^. Furthermore, cadmium and lead disrupt thyroid homeostasis^47^, whereas mercury decreases heart rate variability (HRV)^48^, which is linked to emotional self-regulation^49^. Interestingly, the levels of adrenaline, noradrenaline, and dopamine in serum and urine *increase* with cadmium and mercury exposure due to the inactivation of the catecholamine-degrading enzyme catechol-O-methyl transferase (COMT)^47^.

### Microbiome and heavy metals

The gut microbiome can alter the metabolic outcomes of heavy metals, and in turn, heavy metals influence the viability and metabolism of the microbiome^50^. In their review, Giambo and colleagues^51^ reported that exposure to metals such as arsenic, cadmium, lead, and mercury can change the composition, diversity, homogeneity, and structure of the gut microbiota. They found that higher levels of arsenic in humans are associated with decreased levels of *Bacteroides* and *Bifidobacterium*, while exposure to cadmium, mercury, lead and arsenic is linked to an increase in *Proteobacteria* and pathogenic bacteria. Conversely, various species of *Lactobacillus* and *E. coli* can hinder the absorption of heavy metals; *Lactobacillus* does this by binding heavy metals to their surface, while *E. coli* ingests them^49^. Moreover, probiotics such as *Faecalibacterium*, *Akkermansia muciniphila*, *Bacillus cereus*, and *Lactobacillus rhamnosus* exhibit protective effects against arsenic and cadmium exposure, confirming the essential role of the intestinal microbiome in limiting the uptake of and facilitating the removal of heavy metals from the body^52^.

### Executive functions, mental health, and behaviour

The term “executive functions”, often equated with self-control or cognitive control, refers to processes of control and regulation that enable goal-directed, flexible, and situation-adaptive actions or behaviours. These functions are primarily engaged in situations that require deviation from habitual actions^53^. While the literature discusses a variety of complex and higher-level executive functions (e.g., reasoning, problem solving, and planning), three fundamental executive functions can be used to explain (abnormal) behaviours: updating, inhibition, and shifting^54^. Updating describes the ability to maintain information in working memory over a specific period (e.g., during an action, a conversation, or a cognitively demanding task) and to refresh this information as necessary. When this executive function is impaired, individuals may quickly forget things, lose the thread of a conversation, become scatterbrained or absent-minded, and be unable to register (situational) changes in their immediate environment (“environmental [un]awareness”^55^). Inhibition refers to the suppression of inappropriate responses and is necessary for controlling not only cognitive responses (inhibition of thoughts) but also emotional responses (inhibition of emotional impulses, cf. effortful control) and motor responses (inhibition of inappropriate movements). When inhibition fails, individuals may struggle to stop disruptive thoughts or restlessness and/or exhibit impulsive and heightened emotional reactions. Shifting is the ability to transition between different tasks or topics. Individuals who experience impairments in shifting often find it difficult to escape from ruminative thoughts and have trouble adapting to changes in their daily lives. Numerous studies have established substantial links between executive functions, appropriate social behaviours, academic achievement, and career success^56^. Impaired executive functions are observed in individuals with ADHD^57^, autism^58^, epilepsy^59^, mood and depressive disorders^60^, and burnout syndrome^61^, as well as in prisoners^62^ and individuals with chronic diseases such as chronic fatigue syndrome (CFS)^63^, fibromyalgia^64^ and post-COVID syndrome^65^. While significant attempts have been made to pinpoint the mechanisms governing executive functions within specific brain regions, these efforts have been largely unsuccessful^66^. Intriguingly, recent findings regarding the microbiome suggest that executive functions might also be influenced by microbiome-associated metabolites, lending credence to the emerging hypothesis of a gut–brain connection^67,68^. Nonetheless, to date, no empirical studies have explored the extent to which microbiome-associated metabolites may contribute to variations in executive functions within the context of daily life^69^.

### The present study

On the basis of the assumption that behavioural anomalies, such as autism, antisocial disorders, and ADHD, are merely extreme variations of normal behaviour and do not represent qualitatively distinct behaviours, the aforementioned considerations led to the hypothesis that imbalances in catecholamines, microbiome-associated metabolites, and heavy metal exposure are not only relevant to these disorders but may also function as potential biological markers for variations in social behaviours, executive functions, inattention, hyperactivity, and impulsivity (ADHD symptoms) among typically developing school children. It was hypothesized that the variance in social behaviours, executive functions, and ADHD symptoms within the typical behavioural range can be explained by variations in microbiome-associated catecholamine precursor metabolites, the noradrenaline/adrenaline ratio, and the heavy metal load. Additionally, this research aimed to determine whether a highly altered microbiome-associated catecholamine pathway, a low noradrenaline/adrenaline ratio, and heavy metal overload occur significantly more frequently in children exhibiting the most impaired behaviours than their peers.

## Methods

### Participants and procedure

The 87 children (49 boys, 38 girls) included in this study initially participated in a longitudinal survey aimed at identifying common roots of problems in the development of mathematical and literacy skills and were allowed by parents to additional participate in the present study. At the first measurement point, participants who attended regular primary schools in urban and rural areas in Germany were aged between eight years and nine months to ten years (*M*_*age*_ = 9.4 years, *SD* = 0.30). The majority of them (94%) were German native speakers. Approximately one-third of the participants hailed from households with medium educational attainment (upper secondary school certificate: mothers 36,6%, fathers 23,1%) or low educational levels (lower secondary school certificate: mothers 1,2%, fathers 11,5%). In contrast, nearly two-third represented households with high educational levels (higher secondary school diploma, university degree, or doctoral degree: mothers 62,2%, fathers 65,4%), indicating that individuals from these households were overrepresented in this study. To assess microbiome-associated metabolites, the noradrenaline/adrenaline ratio and heavy metal load, a ready-to-use test kit was sent to the parents. They received detailed instructions to collect two urine samples from their child on any day during a six-week summer vacation and to subsequently send these samples to the laboratory (first measurement point, T_1_, end of Grade 3). Three months later (second measurement point, T_2_, Grade 4), questionnaires regarding the children’s ADHD symptoms, executive functions and social behaviours were completed by their parents and primary school teachers. Teacher questionnaires were completed for 59 children (68%), and parent questionnaires were completed for 81 children (93%).

Additionally, the study gathered information from parents regarding cesarean deliveries, neurodevelopmental abnormalities, and regular medication use. Subsequent analyses indicated that these factors did not significantly affect the urinary results. These findings are reported exclusively in the Supplementary Information.

### Ethical approval

All procedures and experimental protocols performed in this study were approved and were in accordance with the relevant guidelines, regulations and ethical standards of the institutional review board of the University of Education of Ludwigsburg. Informed consent was obtained from all parents of the participating children.

### Physiological and behavioural assessments

To investigate the biological markers, microbiome-associated metabolites and heavy metal load were measured from the first morning urine sample and catecholamines were measured from the second morning urine sample (both midstream urine). For one of the children, parents did not collect the second sample.

### Microbiome-associated metabolites of hormone precursors

The metabolites of gut-associated hormone precursors were assessed by measuring the urinary levels of several organic acids. These acids are produced by gut bacteria from phenylalanine, tyrosine and L-dopa, which are essential for the synthesis of catecholamines and, in part, for thyroid hormone synthesis^70^. 3-Phenylpropionic acid (μg/g creatinine) and *p*-OH-phenylacetic acid (mg/g creatinine) are products resulting from the bacterial degradation of unabsorbed phenylalanine in the intestinal lumen^71,72^, indicating potential overgrowth of *Bacteroides fragilis*^73^ or *Giardia lamblia*^34^ or increased intestinal breakdown of phenylalanine^74^. 4-Hydroxybenzoic acid (mg/g creatinine) is a degradation product of the amino acid tyrosine^75^, which, like phenylalanine, serves as a precursor for catecholamines as well as the thyroid hormone thyroxine. Elevated levels of this acid indicate potential overgrowth of *E. coli* strains^76^ and increased breakdown of tyrosine^74^. Furthermore, the organic acid 3,4-dihydroxyphenylpropionic acid (μg/g creatinine) denotes an increased breakdown of L-dopa, which is a precursor for catecholamines but not for thyroxine^77^. It also serves as a marker for higher production levels of intestinal *Clostridia* and *Pseudomonas* strains^71^. To calculate the additive load of these organic acids, a sum score was computed by summarizing the standardized *z* scores of the four organic acids. Higher scores signify a greater load of organic products, which may indicate increased intestinal breakdown of catecholamine and thyroxine precursors or dysfunction in their synthesis pathways. For a detailed description of the urine analysis procedures conducted in the laboratory, see the Supplementary Information.

### Heavy metal load

The levels of arsenic, lead, cadmium and mercury (each reported as μg/g creatinine) were determined from the same urine samples. To compute the additive load, the sum score was calculated by summarizing the standardized *z* scores of each heavy metal. For details regarding the urine analysis procedures conducted in the laboratory, see the Supplementary Information.

### Noradrenaline/adrenaline ratio

The amounts of noradrenaline and adrenaline (each expressed as μg/g creatinine) were measured from the second morning urine samples. The ratio of noradrenaline to adrenaline was calculated to determine the balance between these two catecholamines. For detailed descriptions of the urine analysis procedures conducted in the laboratory, see the Supplementary Information.

### Social behaviour

Social behaviours and behavioural problems were assessed in Grade 4 via two teacher questionnaires. First, the teachers completed the Teacher Checklist of Social and Learning Behaviour (in German: “Lehrereinschätzliste für Sozial- und Lernverhalten”, *LSL*^78^*,* Cronbach’s alpha* r* = 0.96) to evaluate various aspects of the children’s social behaviour—namely, cooperation (e.g., making compromises), self-perception (e.g., recognizing their own mistakes in disputes), self-control (e.g., delaying gratification), empathy and helpfulness (e.g., proactively assisting others), appropriate assertiveness (e.g., resolving disputes nonviolently) and social contact (e.g., expressing appreciation for others). The LSL sum score was calculated as the total score of the six subscales (maximum score: 90). Second, the three scales related to social behaviour from the German teacher version of the Strengths and Difficulties Questionnaire (*SDQ*^79^, Cronbach’s alpha *r* = 0.84*;* maximum score per scale: 15) were employed to assess conduct problems (e.g., frequently fighting with peers), peer problems (e.g., being picked on or bullied by peers), and prosocial behaviour (e.g., often volunteering to help others). The SDQ sum of social behavioural problems was calculated by scoring problems positively and strengths negatively. The final sum score of social behaviour was generated by subtracting the *z* score of the SDQ sum from the LSL *z* score, with a higher score indicating more socially appropriate behaviours.

### Basic executive dysfunctions

Parents completed the German version of the Behaviour Rating Inventory of Executive Function (BRIEF^80^, Cronbach’s alpha* r* = 0.91) to evaluate their child’s inhibition (e.g., interrupting others; losing control of emotions or behaviours more easily than peers), shifting (e.g., resistance to or difficulty accepting friends, chores, alternative approaches to solve schoolwork problems, etc.; excessive rumination on a particular topic), and working memory as an indicator for updating (e.g., forgetfulness regarding information retrieval or difficulties in completing tasks that require more than one step) in daily life (maximum score: 84). The sum score was calculated from the three subscales, with a higher score reflecting poorer executive functioning.

### ADHD symptoms

Parents assessed inattention (e.g., often seeming not to listen when addressed), impulsivity (e.g., frequently blurting out answers before questions are finished), and hyperactivity (e.g., frequently fidgeting with hands and feet or sliding around in the chair),—which are the cardinal symptoms of ADHD,—via the German ADHD rating scale (*FBB-ADHS*^81^, Cronbach’s alpha *r* = 0.90). This questionnaire consists of 20 items assessing the severity and perceived impact of the cardinal symptoms as defined by the ICD-10 and DSM-5. The sum score was calculated as the total of the three subscales (maximum score: 60), with a higher score indicating behaviours more indicative of ADHD.

### Statistical analysis

All the statistical analyses were performed via the IBM SPSS Statistics and AMOS software packages (Version 22.0; SPSS, Inc., New York, USA, 2013). A boy with outlier values in microbome-associated metabolites and behavioural scales (see descriptives in Table 1) was excluded from correlation and path analyses because his inclusion resulted in an overestimation of the correlations and paths between several variables. To determine the statistical relationships between biological and behavioural markers, two-tailed bivariate Pearson product‒moment correlation coefficients were calculated. Path analyses were subsequently performed to evaluate the relationships among the variables in the model simultaneously. In these analyses, physiological variables (measured at T_1_) were intercorrelated, and paths were set from these variables to the behavioural variables measured at T_2_, with *p* values of 0.05 or less considered statistically significant. Owing to missing values in the returned teacher and parent questionnaires, a complete dataset was obtained for only 54 children. Analysing the data with this limited sample would have resulted in reduced statistical power and potential bias in the interactions between physiological variables. Therefore, analyses were conducted via the full information maximum likelihood method in AMOS (FIML; Analysis of Moment Structures; Arbuckle, 2013) to estimate parameters from all the children’s data (excluding the outlier boy), regardless of missing data points. Finally, classification analyses were performed to assess whether the quarter of children with the most reduced social behaviours (i.e., those with a social behaviour score at or below the 25th percentile), the quarter with the most impaired executive functions (i.e., those with an executive dysfunctioning score at or above the 75th percentile), and the quarter with the highest scores in ADHD symptoms (i.e., those with an ADHD score at or above the 75th percentile) corresponded with those exhibiting the most microbiome-associated metabolites, the lowest noradrenaline/adrenaline ratio or the highest heavy metal load.

**Table 1:** Descriptives and correlations between biological and behavioural data (outlier excluded from correlation analysis).

		Correlations														Descriptives					
	Variable	1	2	3	4	5	6	7	8	9	10	11	12	13	14	N	Min	Max	Outlier	M	SD
1	Microbiome-associated metabolites T_1_															86	− 3.5	8.7	14.5	− 0.06	2.517
2	Noradrenaline/Adrenaline Ratio T_1_	− .09														85	1.8	27.0	11.9	6.32	3.972
3	Heavy metal load T_1_	.33**	.23*													86	− 2.3	9.5	0.0	0.07	2.171
4	ADHD symptoms^1^ T_2_	.37**	.01	.04												80	0	31	53	8.37	6.525
5	Executive dysfunctioning^1^ T_2_	.31**	− .05	.04	.77**											80	28	62	81	41.03	7.818
6	Social behaviour T_2_	− .43**	.17	− .49**	− .39**	− .45**										58	− 5.8	1.9	− 5.3	0.14	1.767
	*Single values microbiome-associated metabolites T*_*1*_*:*																				
7	3-phenylpropionic acid^2^	.75**	.00	.50**	.16	.17	− .38**									86	0.01	0.08	0.04	0.028	0.013
8	*p*-OH-phenylacetic acid^3^	.68**	− .17	.05	.31**	.21*	− .34**	.34**								86	4.50	65.47	24.68	13.152	8.031
9	4-hydroxybenzoic acid^3^	.70**	− .06	.20	.24*	.19	− .21	.30**	.26*							86	0.14	4.99	3.42	0.783	0.629
10	3,4-dihydroxyphenylpropionoic acid^2^	.63**	− .02	.13	.35**	.33*	− .03	.39**	.20	.40**						86	11.67	277.74	660.83	63.860	45.625
	*Single values heavy metals T*_*1*_*:*																				
11	Arsenic^2^	.10	.11	.47**	− .02	.02	− .43**	.10	.00	.20	− .06					86	2.0	144.3	6.0	13.63	22.917
12	Lead^2^	.33**	.16	.57**	.09	.07	− .26	.43**	.07	.18	.23*	− .00				86	0.2	5.3	0.8	0.76	0.644
13	Cadmium^2^	.34**	.15	.56**	.04	− .02	− .38**	.49**	.15	.11	.14	.01	.17			86	0.1	3.3	0.6	0.40	0.362
14	Mercury^2^	− .09	.04	.45**	− .04	− .01	− .11	.00	− .12	− .08	− .04	− .06	− .01	− .03		86	0.0	33.8	0.6	1.90	4.487
	*Single values catecholamines T*_1_:																				
	Noradrenaline^2^	.16	.26*	− .09	.12	− .10	.01	.20	.13	.08	− .17	.05	− .14	.20	− .14	58	17.23	88.41	102.05	41.00	14.446
	Adrenaline^2^	.21	− .65**	− .19	.07	.08	− .22	.06	.34**	.18	− .33*	− .02	− .16	.06	− .19	58	1.95	28.29	8.56	8.50	4.457
	*Subscales ADHD symptoms T*_2_*:*																				
	scale Unattention	.32**	.00	.03	.90**	.76**	− .42**	.15	.35**	.17	.23*	.00	.06	− .02	.02	80	0	15	24	5.47	3.631
	scale Hyperactivity	.31**	.05	.00	.87**	.50**	− .18	.12	.20	.22*	.39**	− .02	.05	.03	− .10	80	0	13	20	1.65	2.605
	scale Impulsivity	.28*	− .01	.13	.69**	.65**	− .28*	.15	.15	.25*	.28*	− .07	.17	.19	− .05	80	0	6	9	1.25	1.436
	*Subscales executive dysfunctioning T*_2_:																				
	Updating/Working memory^1^	.40**	− .08	.08	.69**	.85**	− .33*	.26*	.29*	.23*	.37**	.08	.06	− .01	.06	80	10	26	29	15.56	4.161
	Inhibit^1^	.16	.00	.06	.70**	.72**	− .37**	.03	.11	.14	.21	.00	.13	.02	− .05	80	10	24	30	13.94	3.281
	Shift^1^	.09	− .01	− .09	.32*	.72**	− .24	.06	.05	.02	.14	− .05	− .03	− .07	− .05	80	8	22	22	11.53	2.678
	*Subscales social behaviour T*_2_:																				
	LSL, Positive social behaviour^4^	− .39**	.17	− .45**	− .44**	− .53**	.95**	− .35**	− .37**	− .13	.03	− .32*	− .18	− .35**	− .22	58	42	90	48	78.56	10.901
	… Scale Cooperation	− .42**	.12	− .39**	− .36*	− .42**	.82**	− .40**	− .42**	− .12	.07	− .23	− .20*	− .40**	− .15	58	5	15	8	13.58	2.200
	… Scale Sense of self	− .35**	.07	− .32**	− .36*	− .42**	.73**	− .25*	− .34**	− .12	− .11	− .15	− .12	− .25*	− .23	58	7	15	9	13.10	2.049
	… Scale self-control	− .27*	.11	− .24	− .52**	− .43**	.71**	− .10	− .28*	− .23	.06	− .38**	− .02	− .10	− .12	58	6	15	9	12.47	2.386
	… Scale empathy and helpfulness	− .21	.20	− .35**	− .18	− .38**	.68**	− .34**	− .23	.11	− .01	− .17	− .22	− .34**	− .13	58	6	15	2	12.84	2.668
	… Scale Appropriate assertiveness	− .34**	.14	− .44**	− .42**	− .41**	.83**	− .28*	− .27*	− .24	.11	− .32*	− .17	− .28*	− .25	58	5	15	10	13.34	2.268
	… Scale Social contacts	− .30*	.16	− .43**	− .32*	− .47**	.86**	− .32*	− .27*	− .08	.01	− .32*	− .17	− .32*	− .21	58	7	15	10	13.22	1.947
	SDQ, Social behavioural problems^1,5^	.44**	− .16	.48**	.31*	− .36*	− .96**	.38**	.29*	.26	.08	.50**	.30*	.37**	.01	58	− 5	11	10	1.71	3.799
	… Scale Conduct problems	.38**	− .13	.16	.30*	.25	− .71**	.22	.35**	.25	.02	.22	.13	.22	− .10	58	5	11	8	5.76	1.380
	… Scale Peer problems	.31*	− .17	.45**	.12	.29*	− .75**	.28*	.12	.23	.15	.63**	.30*	.27*	− .09	58	5	14	8	5.87	1.698
	… Scale Prosocial behaviour^6^	− .34**	.08	− .46**	− .33	− .31*	.78**	− .37**	− .24	− .14	− .01	− .29*	− .27*	− .37**	− .18	58	9	15	6	13.34	1.771

The relationships between the variables are visually represented in the Supplementary Information via scatter plots (Supplementary Figs. 1 to 4). * p < .05, ** p < .01; T_1_--1st measurement point (Grade 3), T_2_--2nd measurement point (Grade 4).

N—number of children without the outlier; Min—minimum score; Max—maximum score; M—mean; SD—standard deviation; Outlier—values for a boy (according to parents, a child with ADHD) excluded from the analysis because of outliers in microbiome-associated hormone precursor metabolites (sum score and 3,4-dihydroxyphenylpropionoic acid), catecholamines (noradrenaline), executive dysfunction (sum and subscale scores) and social behaviour (subscales empathy and helpfulness; prosocial behaviour);

^1^the higher the score, the more impaired; ^2^μg/g creatinine; ^3^mg/g creatinine;

^4^LSL = Teacher Checklist of Social and Learning Behaviour (“Lehrereinschätzliste für Sozial- und Lernverhalten “, Petermann & Petermann, 2006);

^5^SDQ = Strengths and Difficulties Questionnaire (Goodman, 2005), with reversed polarity included in the social behaviour sum calculation (computed as LSL minus SDQ);

^6^included in the SDQ sum calculation with reversed polarity.

## Results

### Descriptive statistics and correlations of physiological and behavioural variables

Table 1 presents an overview of descriptive statistics for the participants included in the analyses, detailing the minimum, maximum, mean, and standard deviation for each investigated variable, as well as outlier values from the boy excluded from the analysis. It also includes Pearson correlations between the total and individual scores for the examined variables.

Moderately negative correlations were observed between social behaviours and microbiome-associated metabolites (*r* = − 0.43, *p* < 0.001) and between social behaviours and heavy metal load (*r* = − 0.49, *p* < 0.001). These findings suggest that social behaviour is negatively related to both microbiome-associated metabolites and abnormal concentrations of heavy metals; i.e., the greater the load of microbiome-associated metabolites and heavy metals is, the lower the social behaviour. This was particularly evident for the levels of arsenic (*r* = − 0.43, *p* < 0.001) and cadmium (*r* = − 0.38, *p* = 0.003). A similar correlation was found between social behaviours and executive dysfunction (*r* = − 0.45, *p* < 0.001). There was a strong correlation between ADHD symptoms and executive dysfunction (*r* = 0.77, *p* < 0.001). While neither executive dysfunction nor ADHD symptoms correlated with heavy metal load (*r* = 0.04, *p* = 0.753 and *r* = 0.04, *p* = 0.708, respectively), they were associated with increased microbiome-associated metabolites (*r* = 0.31, *p* = 0.005 and *r* = 0.37, *p* < 0.001, respectively), which was particularly evident in updating/working memory (*r* = 0.40, *p* < 0.001). Additionally, microbiome-associated metabolites and heavy metal load were moderately correlated (*r* = 0.33, *p* = 0.002), primarily due to contributions from lead (*r* = 0.33, *p* = 0.002), cadmium (*r* = 0.34, *p* = 0.002) and 3-phenylpropionic acid (*r* = 0.50, *p* < 0.001). The noradrenaline/adrenaline ratio was significantly correlated with only the heavy metal load (*r* = 0.23, *p* = 0.039). The relationships between these variables are also illustrated via scatterplots (see Supplementary Figs. 1 to 4).

### Interplay between microbiome-associated metabolites, heavy metal load, social behaviours, and executive functions

The analyses above revealed that physiological data were moderately correlated with social behaviours, with microbiome-associated metabolites also showing a correlation with executive functions and ADHD symptoms. A path model was subsequently developed to evaluate the impact of microbiome-associated metabolites, the noradrenaline/adrenaline ratio, and heavy metal load on social behaviours, ADHD symptoms and executive functions while considering all the variables simultaneously. To enhance readability and clarity, the polarities of paths related to social behaviours were reversed from negative to positive; simultaneously, “social behaviours” were rephrased as “reduced social behaviours”.

**Fig. 1:**
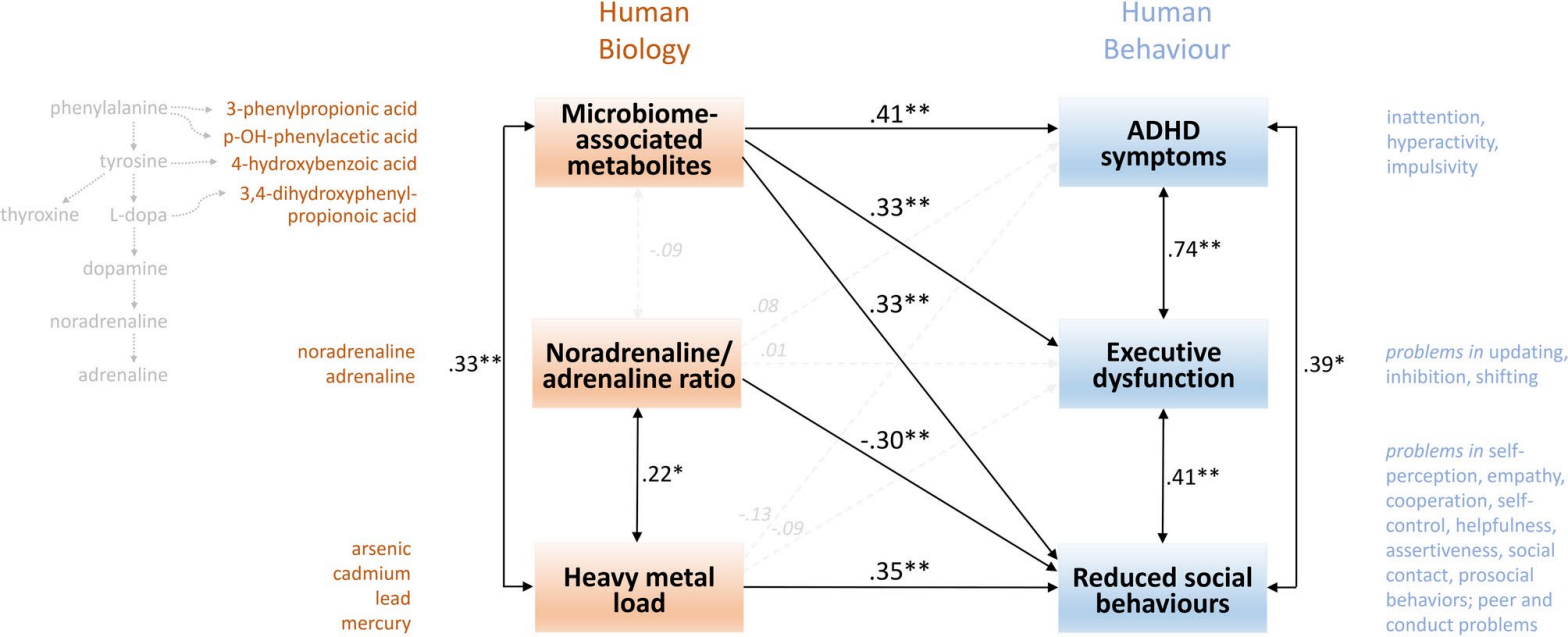
Results of the path analysis: Prediction of executive dysfunction, ADHD symptoms, and reduced social behaviours in typical primary school children from microbiome-associated metabolites of hormone precursors, the noradrenaline/adrenaline ratio, and heavy metal load, using the indicators displayed in orange and blue text on the left and right sides. All the significant paths are in a range between *β* = .30 and *β* = .41 (significance ** *p* < .01, * *p* < .05), indicating that each specific biological marker accounts for 9.0–16.8% of the variance in the specific behavioural factor. While microbiome-associated metabolites predict substantial variance in all three behavioural factors (executive dysfunction, ADHD symptoms and reduced social behaviours), heavy metal load and the noradrenaline/adrenaline ratio account for only reduced social behaviours. Specifically, the higher the load of microbiome-associated metabolites, the greater the incidence of ADHD symptoms, executive dysfunction and reduced social behaviours. Additionally, the higher the heavy metal load and the lower the noradrenaline/adrenaline ratio are, the more conspicuous the social behaviours. In summary, the biological markers accounted for 32% of the interindividual differences in social behaviours, 17% of the ADHD symptoms and 11% of the executive functions in typical primary-school children.

As illustrated in Fig. 1, when all six variables were considered simultaneously, the regression weights remained substantial, which is consistent with the correlations displayed in Table 1. Microbiome-associated metabolites accounted for 10.9% of executive dysfunction (*β* = 0.33, *p* = 0.004), 16.8% of ADHD symptoms (*β* = 0.41, *p* < 0.001), and 10.9% of reduced social behaviours (*β* = 0.33, *p* = 0.003). Although microbiome-associated metabolites were positively correlated with heavy metal load (10.9% shared variance,* r* = 0.33, *p* = 0.004), heavy metal load additionally predicted 12.3% of reduced social behaviours (*β* = 0.35, *p* = 0.002) but did not predict executive dysfunction (*β* = -0.09, *p* = 0.460) or ADHD symptoms (*β* = − 0.13, *p* = 0.259). Similarly, the noradrenaline/adrenaline ratio did not predict executive dysfunction (*β* = 0.01, *p* = 0.948) or ADHD symptoms (*β* = 0.08, *p* = 0.461) but accounted for 9.0% of social behaviours (*β* = − 0.30, *p* = 0.004). Notably, executive dysfunction exhibited substantial variance with ADHD symptoms (54.8%; *r* = 0.74, *p* < 0.001), as did reduced social behaviours (16.8%; *r* = 0.41, *p* = 0.003), and the latter was correlated with ADHD symptoms (15.2%; *r* = 0.39, *p* = 0.005).

### Classification of children with the most conspicuous behaviours

Classification analyses were performed to ascertain whether the children displaying the most reduced social behaviours, the most ADHD symptoms, and the most impaired executive functions were the same individuals who presented the highest levels of microbiome-associated metabolites, the lowest noradrenaline/adrenaline ratio, or the highest heavy metal load. Table 2 presents the classification results in the middle and the statistical values on the right. For example, classification revealed that eight out of 15 children with the most reduced social behaviours concurrently presented high heavy metal loads, whereas 37 children presented neither reduced social behaviours nor high heavy metal loads. The odds ratio indicates how well the group of children with the most reduced social behaviour can be described by a high load of microbiome-associated metabolites, a low noradrenaline/adrenaline ratio and/or a high heavy metal load. The highest odds ratio was found for heavy metal load among children with the most reduced social behaviours, indicating that heavy metal overload occurred six times more frequently in these children than in children with better social skills (odds ratio = 6.04). In contrast, the odds of a high load of microbiome-associated metabolites were 3.40 times higher and for a low noradrenaline/adrenaline ratio 1.94 times higher for these children than in children with better social skills. Furthermore, a high load of microbiome-associated metabolites was found 3.02 times more often in children with the most impaired executive functions than in those with better executive functions and 2.02 times more often in children who exhibited the most ADHD symptoms than in those with very few or no symptoms. In contrast, the heavy metal load and noradrenaline/adrenaline ratio did not significantly differ between children with impaired and better executive functioning or between those with the most and few ADHD symptoms (with odds ratios for both close to one).

**Table 2:** Results of classification analyses for the quarter of typical school children with the highest scores in ADHD symptoms (inattention, hyperactivity, and impulsivity), the lowest scores in social behaviours and the greatest impairment in executive functions.

		Behaviour (Grade 4)						
		ADHD symptoms						
Biological marker (Grade 3)		Increased (PR ≥ 75)	Normal/low (PR < 75)	Sum	Sensitivity	Specificity	Odds ratio	CI 95% odds ratio
Microbiome-associated metabolites	High (PR ≥ 75)	8	14	22	38.1	76.7	2.02	[0.70,5.86]
	Normal/low (PR < 75)	13	46	59				
	Sum	21	60	81				
Noradrenaline/adrenaline ratio	Low (PR ≤ 25)	5	15	20	23.8	74.6	0.92	[0.29, 2.93]
	Normal/high (PR > 25)	16	44	60				
	Sum	21	59	80				
Heavy metal load	high (PR ≥ 75)	5	13	18	23.8	78.3	1.13	[0.35,3.67]
	Normal/low (PR < 75)	16	47	63				
	Sum	21	60	81				

		Executive functioning						
		Decreased (PR ≥ 75)	Normal/low (PR < 75)	Sum	Sensitivity	Specificity	Odds ratio	CI 95% odds ratio
Microbiome-associated metabolites	High (PR ≥ 75)	9	13	22	45.0	78.7	3.02	[1.03,8.84]
	Normal/low (PR < 75)	11	48	59				
	Sum	20	61	81				
Noradrenaline/adrenaline ratio	Low (PR ≤ 25)	5	15	20	25.0	75.0	1.00	[0.31, 3.22]
	Normal/high (PR > 25)	15	45	60				
	Sum	20	60	80				
Heavy metal load	High (PR ≥ 75)	4	14	18	20.0	77.0	0.84	[0.24,2.92]
	Normal/low (PR < 75)	16	47	63				
	Sum	20	61	81				

		Social behaviour						
		Decreased (PR ≤ 25)	Normal/high (PR > 25)	Sum	Sensitivity	Specificity	Odds ratio	CI 95% odds ratio
Microbiome-associated metabolites	High (PR ≥ 75)	7	9	16	46.7	79.5	3.40	[0.97,11.89]
	Normal/low (PR < 75)	8	35	43				
	Sum	15	44	59				
Noradrenaline/adrenaline ratio	Low (PR ≤ 25)	5	9	14	33.3	79.5	1.94	[0.53, 7.13]
	Normal/high (PR > 25)	10	35	45				
	Sum	15	44	59				
Heavy metal load	High (PR ≥ 75)	8	7	15	53.3	84.1	6.04	[1.65,22.09]
	Normal/low (PR < 75)	7	37	44				
	Sum	15	44	59				

PR—percentile rank, CI—confidence interval.

## Discussion

Recent studies have provided substantial evidence that the gut microbiome and associated metabolites play crucial roles in physical and mental health, as well as in various disorders. Previous studies have also reported associations between neurobehavioural disorders and heavy metal overload, as well as imbalanced neurotransmitters. However, these earlier studies did not comprehensively consider all three physiological aspects when predicting mental health. Furthermore, such biological markers are often collected through blood or stool samples, which can be more invasive or less discreet than a urine test. Finally, previous studies were primarily conducted as animal or clinical human studies, limiting their applicability to the behaviour of healthy humans. A novel aspect of the present study is that investigating microbiome-associated metabolites highlights a possible bridge through which the gut is linked with brain function. Specifically, by investigating gut-associated metabolites, the extent to which the microbiota might intervene in the synthesis of hormones and neurotransmitters that play a pivotal role in brain function can be examined. In light of this, the primary objective of the present study was to investigate the load of microbiome-associated metabolites of catecholamine and thyroid hormone precursors, the noradrenaline/adrenaline ratio, the heavy metal load, and their associations with the behaviour and executive functions of typical schoolchildren simultaneously.

Path analysis revealed moderate correlations between social behaviours and executive functions^82^, as well as with ADHD^83^, and demonstrated a strong correlation between executive functions and ADHD symptoms. These findings indicate that reduced social behaviours are associated with diminished executive functions and heightened ADHD symptoms. As expected, even in this sample of typical schoolchildren, the physiological measures proved to be reliable as biomarkers of how socially appropriate a child behaved. This underscores the importance of using urine samples as a non-invasive biological approach for predicting social behaviour. In the path analysis, differences in microbiome-associated metabolites accounted for 17% of the variance in ADHD symptoms and 11% of the variance in executive functions that underlie situationally adaptive actions and self-controlled behaviours. Additionally, nearly one-third of the variance in social behaviours was predicted by considering the load of microbiome-associated metabolites, the noradrenaline/adrenaline ratio and the heavy metal load. This suggests that the greater the overload of heavy metals and microbially associated metabolites of hormone precursors in children and the lower their noradrenaline/adrenaline ratio are, the less socially they behave. Moreover, the greater the child’s burden of microbially associated metabolites is, the greater the degree to which the child displays ADHD symptoms, along with impairments in executive functions, particularly in updating. These findings emphasize the importance of the interaction between the gut microbiota and the biosynthesis of stress hormones such as dopamine, noradrenaline, adrenaline, and the thyroid hormone thyroxine for the child’s behaviour.

The relationships between behaviours, microbiome-associated metabolism, and heavy metal load were observed not only in the overall sample but also in three subsets of children exhibiting the most pronounced behavioural issues: those with the most impaired social behaviour, those with the most significant executive function deficits, and those with the most ADHD symptoms. Compared with their peers, the odds of a high load of microbially associated metabolites were approximately 200% greater for children displaying reduced social behaviours (OR=3.40) or executive function impairments (OR=3.02) and 102% greater for those with a high level of ADHD symptoms (OR=2.02). This underscores the importance of considering alterations in the microbiome-associated catecholamine pathway in behavioural anomalies. Finally, a particularly important role for heavy metal exposure was identified. The odds of being burdened with a high heavy metal load were 504% higher in the group of children with the most pronounced social behavioural issues (OR=6.04), indicating that heavy metal overload was even more common in these children than a high load of microbiome-associated metabolites. This strongly suggests that it is imperative to further investigate the role of bodily levels of arsenic, cadmium, and lead in social behavioural anomalies.

Nonetheless, moderate correlations were observed between heavy metal load and microbiome-associated catecholamine precursor metabolites; higher levels of metabolites, particularly 3-phenylpropionic acid, were associated with increased heavy metal load. This aligns with the notion that the intestinal microbiome and heavy metals interact; for example, the microbiota can form protective barriers that inhibit the entry of toxins into the body, whereas heavy metals can influence the viability and metabolism of the microbiome^2,50^.

### Implications and future research

How should one address the significant burden of heavy metals and the increased intestinal degradation of amino acids that are essential for hormone synthesis, particularly in the context of behavioural problems? A recent review by Kothapalli^84^ emphasized the urgent need for detoxification therapies to prevent children and adults from experiencing heavy metal-induced neurotoxicity and subsequent damage to the central nervous system. The findings reported in the present study, regarding the association of the heavy metal load range with the behavioural range of typical school children, suggest that such prevention might even be taken into account to ensure normal behavioural development. Several studies have supported the notion that nutritional strategies, such as supplementation with zinc, N-acetylcysteine, fatty acids, or probiotics, as shown below, might serve as a valuable complement in this context. For example, zinc supplementation has been shown to effectively mitigate cadmium toxicity^85^. Second, according to Kelly^86^, N-acetylcysteine may provide another viable approach because of its critical role in endogenous detoxification mechanisms. Research indicates that N-acetylcysteine supplementation also has positive effects on impulsivity and compulsivity^87^, autism^88^, ADHD^89^, schizophrenia, and affective disorders^90^, as well as on gastrointestinal conditions such as Crohn’s disease and ulcerative colitis^91^, thereby reinforcing the importance of the potential of this nutrient. Furthermore, N-acetylcysteine exhibits anti-inflammatory activities and can enhance the immune response^85^, which is particularly noteworthy given that the organic acids produced in the gut—those examined in this study—also possess immune-stimulating effects^36–39^. Third, supplementation with omega-3 fatty acids might aid the microbiome in regulating metabolic imbalances, as these acids are partially produced in a healthy gut to safeguard the intestinal mucosa against inflammation and harmful environments^92^. Fourth, incorporating probiotics, such as *Bifidobacterium*, *Lactobacillus* species, and *Escherichia coli* Nissle 1917, or promoting the growth of *Faecalibacterium* or *Akkermansia muciniphila* might effectively support intestinal metabolism and mitigate the accumulation of heavy metals in the body^93,94^.

Finally, the interaction between individual predispositions and environmental factors should be considered. Children with weakened bodily defences—possibly associated with gastrointestinal or inflammatory diseases, and/ or diminished detoxification capabilities (disposition)—might be more susceptible to accumulating toxic heavy metals in their bodies and developing significant microbiome changes. When these children are additionally exposed to environmental pollutants, their risk of developing behavioural disorders might further increase. Further research is needed to explore the validity of these assumptions.

### Limitations of the study

This pilot study has several limitations. Existing research has underscored the intestinal synthesis of the urinary metabolites investigated in this study, and Daneberga and colleagues cautiously proposed that these urinary metabolites may serve as biomarkers for shifts in the gut microbiota^95^. However, it remains unclear at this stage how much of the urinary metabolites are definitively derived from microbial activity versus how much is merely associated with it. Furthermore, uncertainty exists regarding the relationship between urinary metabolites and their counterparts in stool or blood, as at least amino acids may exhibit differing patterns across these samples^96^. This verification is particularly important given the potential confounding effects of dietary intake and the possibility that the urinary tract flora might influence the results, especially in cases of urinary tract infections. Consequently, further studies are vital to establish whether the investigated metabolites consistently represent reliable biomarkers for the gut microbiome and the specified behavioural variables. To address these issues, additional research is needed to explore the correlations between the gut microbiota and urinary metabolite levels as well as their concentrations in the stool or blood. They may reveal whether the relationships between different biological samples are positive, negative, or uncorrelated due to variations in metabolic pathways. Furthermore, additional influencing factors related to the metabolites under investigation include genotype, nutrition, and energy metabolism^97^. This consideration is equally relevant to the levels of heavy metal excretion measured in this study. Since the study does not delve into these aspects in detail, it remains unclear how the excreted concentrations correlate with the blood and hair levels of heavy metals, as well as the individual capacities for detoxifying these substances. Importantly, no additional information is available regarding sources of heavy metal exposure or demographic factors that could influence the risk of heavy metal accumulation or the neurodevelopmental disorders under investigation. Ultimately, future research should aim to identify these factors and their implications for the results presented here.

## Supplementary Information

Supplementary Information.

## Data Availability

The datasets generated during and/or analysed during the current study are available from the corresponding author upon reasonable request.
