# Supplementary material for: Heavy metals, noradrenaline/adrenaline ratio, and microbiome-associated hormone precursor metabolites: biomarkers for social behaviour, ADHD symptoms, and executive function in children
Source: Sci Rep. 2025 May 30;15:19006. doi: 10.1038/s41598-025-00680-5 (PMC12125380; doi:10.1038/s41598-025-00680-5)
Supplement: Supplementary file 1 — Supplementary Information. [file 41598_2025_680_MOESM1_ESM.pdf]

Supplementary Information

Manuscript Title: Heavy metals, noradrenaline/adrenaline ratio, and microbiome-associated hormone precursor metabolites: biomarkers for social behaviour, ADHD symptoms, and executive function in children

Author: Kristin Krajewski**,** University of Education, Ludwigsburg, Germany

Contents

1. Supplementary statistics:
    - Additional descriptive statistics for the examined sample
2. Supplementary Methods: Details of urinary analyses:
    - Organic acids (microbiome-associated metabolites of hormone precursors)
    - Heavy metal load of arsenic, lead, and cadmium
    - Heavy metal load of mercury
    - Noradrenaline/ adrenaline ratio
3. Supplementary figures (scatter plots):
    - F1: Relationships between noradrenaline/ adrenaline ratio, behavioral scores, microbiome-associated metabolites and heavy metal load
    - F2: Relationships between level of microbiome-associated metabolites, behavioral scores, and heavy metal load
    - F3: Relationships between heavy metal load and behavioral scores
    - F4: Interrelationships within behavioral scores

1. Supplementary Statistics

The following statistics are presented, as the study also collected information regarding cesarean deliveries, some neurodevelopmental abnormalities, and regular medication use via parental surveys. Subsequent analyses revealed that these factors did not have a significant impact on the examined variables (sum scores) and were therefore excluded from further analysis:

- 24/87 (28%) children were reported as *cesarean deliveries.* They did not differ in the microbiome-associated urinary metabolites (sum score) from the children born normally: cesarean (M = 0.51/ SD = 3.11) versus vaginal birth (M = -0.40/ SD = 2.05), no significant difference (T = 1.57, d = 0.35, p = .12).

- 3/87 (3%) children had an *ADHD diagnosis*. 1 of them was excluded from the correlation and path analyses because he had many outlier values (see Table 1). He received Elvanse

medication during school hours, but not during the urine test. The other two children with ADHD showed no outlier values in any of the measured variables and did not receive medication.

- 4/87 (5%) children had *enuresis*. They showed no outlier values in overall scores, but 1 of them was the child with the highest lead level (5.3 µg/g creatinine), another of these 4 children exhibited the lowest NA/AD ratio (1.8).
- 0/87 (0%) children had *epilepsy*.
- 1/87 (1%) child took *L-Thyroxine* and 1/87 (1%) took *Bisoprolol* during urine measurement. Neither of the two children showed outlier values in any of the variables measured.

2. Supplementary Methods: Details of urinary analyses conducted at the Ganzimmun Diagnostics Laboratory in Mainz, Germany

Organic acids measured to determine microbiome-associated metabolites of hormone precursors. The level of organic acids– namely, 3-phenylpropionic acid (PPA), *p*-OH-phenylacetic acid (POP), 4-OH-benzoic acid (4OB), and dihydroxyphenylpropionic acid (DPP) – were measured from the first morning urine samples by LC-MS/MS (HS652 oil pump, autosampler ProStar and Varian 320-MS LC/M; Agilent Technologies, Waldbronn, Germany). Before measurement, each urine sample was diluted 1:10 with acetone. An HPLC column (Atlantis T3, 2.1 x 150 mm, 3 µm; Waters Corporation, Eschborn, Germany) was used to determine POP, 4OB, and DPP levels. Another HPLC column (Pursuit XRs C18, 2.0 x 1,500 mm; Agilent Technologies, Waldbronn, Germany) was used to measure PPA. The other reagents, standard substances, and control materials were purchased from Merck KGaA (Darmstadt, Germany). To control for quality, the investigated organic acids were added to blank (that is, not containing any traces of drugs or medications) urine samples (Medichem Diagnostica GmbH, Steinenbronn, Germany) at two different concentrations and were measured before and after measuring each sample. The measurement results of the quality controls were evaluated using Unity Real Time software (BioRad® Laboratories, Hercules, California, USA) and were allowed to deviate from the target value by a maximum of 20%. Creatinine levels in the urine were determined according to Jaffé's method[2] using an ADVIA Chemistry XPT (Siemens Healthcare, Erlangen, Germany).

Sources for clinical evaluation of the analytes:

Lord, R. S. & Bralley, J. A. Clinical applications of urinary organic acids. Part 2. Dysbiosis markers. *Altern Med Rev*. **13**, 292–306 (2008).

Jaffé, M. Über den Niederschlag, welchen Pikrinsäure in normalem Harn erzeugt und über eine neue Reaktion des Kreatinins. Hoppe-Seylers. Z*. Physiol. Chem.* **10**, 391– 400 (1986).

Heavy metal load of arsenic, lead, and cadmium. Urinary concentrations of arsenic, lead, and cadmium were measured by inductively coupled plasma mass spectrometry (ICP-MS) on an Agilent 8800 instrument (Agilent, Santa Clara, USA) equipped with a Cetac ASX-520 autosampler (Cetac, Omaha, USA). The first morning urine samples were prepared for measurement by performing the following: 1 mL of each urine sample was mixed with 200 µl of nitric acid (69%, analytical grade, Merck KGaA, Darmstadt, Germany) and 50 µl of hydrochloric acid (30%, Suprapur, Merck). Subsequently, 8.75 mL of deionized water (Merck Milli-Q) were added and the solution was homogenized. Calibration of the Agilent 8800 was conducted every workday in a range of 0.1 to 200 µg/L using a 10 mg/L multi-element standard (Calibration Standard 2A, Agilent). For quality control, the heavy metal concentrations in certified freeze-dried urine samples (ClinCheck, Recipe, Munich, Germany) were measured at the beginning and end of each measurement. The validation of this procedure resulted in the following coefficients of variation: 1.9 – 4.3% (As), 1.1 – 12.4% (Pb) and 1.8 – 3.1% (Cd). The limits of detection for this method were 0.015 µg/l (As), 0.24 µg/l (Pb) and 0.024 µg/l (Cd).

Source for clinical evaluation of the analytes:

Thomas, L. *Labor und Diagnose* (6th ed.) (TH-Books, 2005).

Heavy metal load of mercury. Urinary mercury concentration was measured by inductively coupled plasma mass spectrometry (ICP-MS) on a Varian 820 instrument (Varian, Palo Alto, USA) equipped with a Cetac ASX-520 autosampler (Cetac, Omaha, USA). To prepare the samples for measurement, a modified procedure from the literature was conducted: 1 mL nitric acid (69%, analytical grade, Merck) was added to 1 mL of each urine sample. The mixture was heated at 80 °C in a closed vessel for 5 hours. Subsequently, 8 mL of deionized water (Merck Milli-Q) were added and the solution was homogenized. Calibration was conducted every workday in a range of 1 to 16 µg/L using a 1,000 mg/L standard (Merck Certipur). The mercury concentrations of quality control samples prepared from a 1,000 mg/L standard (Sigma Aldrich TraceCERT) were measured at concentrations of 5 and 12 µg/L. The validation of this procedure resulted in a coefficient of variation of 1.4 – 8.0% and a detection limit of 0.2 µg/L.

Sources for clinical evaluation of the analytes:

Wieberneit, N. *Einsatz der ICP-Massenspektrometrie zur Mutlielementbestimmung in biologischen Proben* (Universität Hamburg, 2001).

Goulle, J.P. *et al.* Metal and metalloid multi-elementary ICP-MS validation in whole blood, plasma, urine and hair: Reference values. *Forensic Science International* **153**, 39-44 (2005).

Noradrenaline/ adrenaline ratio. Noradrenaline and adrenaline levels were measured using high-performance liquid chromatography (HPLC) with an electrochemical detector (Hitachi High-Tech, Düsseldorf, Germany; Chromsystems Instruments & Chemicals, Munich, Germany). All columns, reagents, and calibrators were sourced from Chromsystems Instruments & Chemicals (Munich, Germany) and processed according to the provided protocols. Coefficients of variation for each analyte were calculated accross two levels of quality control obtained from Chromsystems Instruments & Chemicals (Munich, Germany). The noradrenaline/adrenaline ratio was determined by dividing the concentration of noradrenaline (µg/L) by the concentration of adrenaline (µg/L). Creatinine levels in urine were assessed using Jaffé's method with the ADVIA Chemistry XPT (Siemens Healthcare, Erlangen, Germany).

References:

Jaffé, M. (1986). Über den Niederschlag, welchen Pikrinsäure in normalem Harn erzeugt und über eine neue Reaktion des Kreatinins. Hoppe-Seylers. Z*. Physiol. Chem.* **10**, 391–400.

Zimmermann, E., Donike, M., Schänzer, W. (1985). Katecholaminspiegel, psychische Aktivierung und Wettkampfstabilität. In: Franz, IW., Mellerowicz, H., Noack, W. (eds) *Training und Sport zur Prävention und Rehabilitation in der technisierten Umwelt / Training and Sport for Prevention and Rehabilitation in the Technicized Environment*. Springer, Berlin, Heidelberg.

3. Supplementary Figures

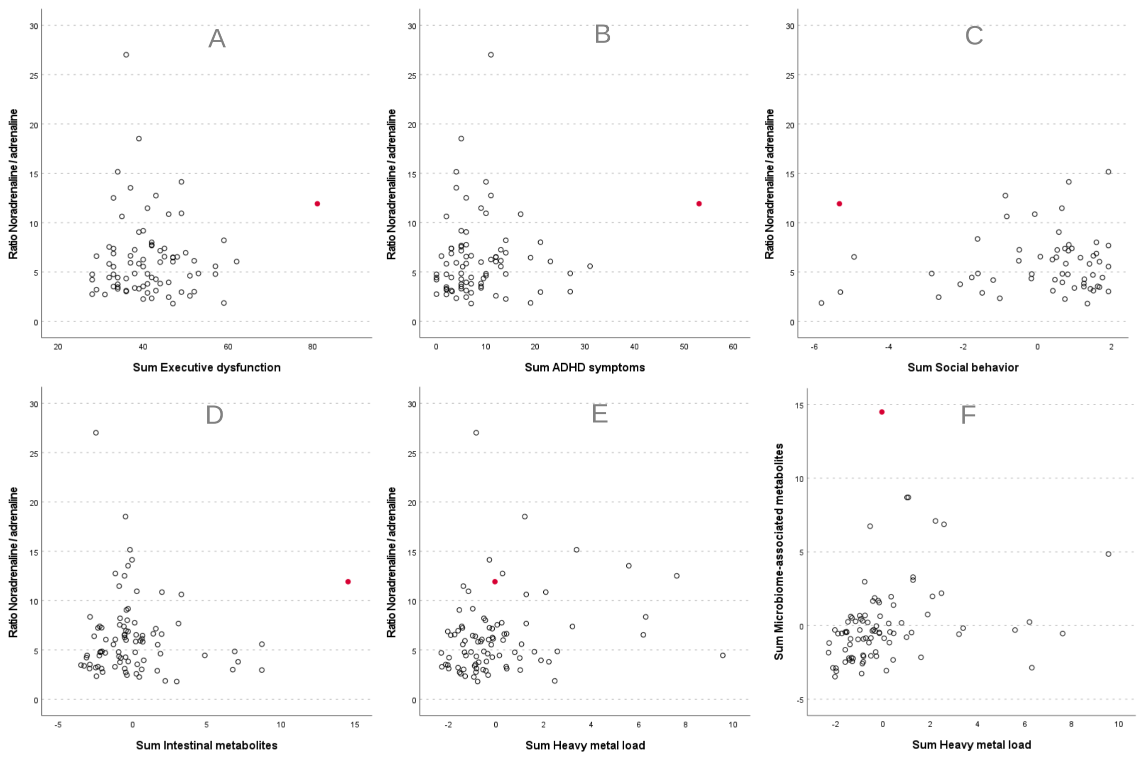

Supplementary Figure 1. Relationships between noradrenaline/ adrenaline ratio, behavioral scores, microbiome-associated metabolites and heavy metal load. The scatter plots illustrate the noradrenaline/ adrenaline ratio in relation to scores in executive functions (A), ADHD symptoms (B), and social behavior (C) as well as microbiome-associated metabolites (D) and heavy metal load (E), as well as the relationship between microbiome-associated metabolites and heavy metal load (F) for each individual child. Each dot represents scores of a child who took part in both measures. Outlier values of the boy excluded from correlation and path analyses are marked by a red dot. In charts A-E, children with a higher noradrenaline/ adrenaline ratio (i.e., relatively higher noradrenaline and lower adrenaline level) are represented by dots that are higher up. Children with worse executive functions (A), more ADHD symptoms (B), or higher heavy metal load (E, F) are represented by dots to the right; children with more unsocial behavior (C), to the left.

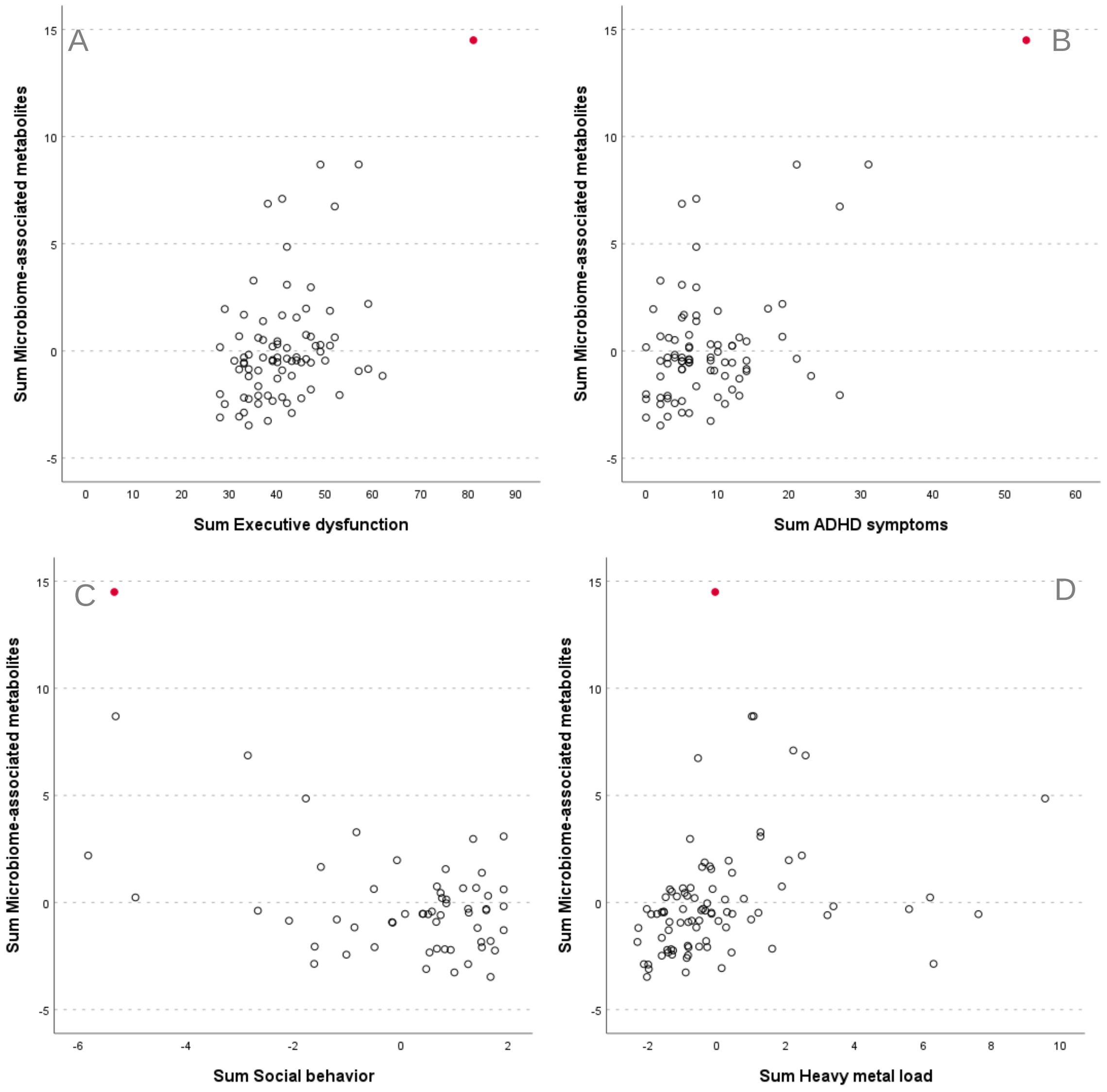

Supplementary Figure 2. Relationships between level of microbiome-associated metabolites, behavioral scores, and heavy metal load. The scatter plots illustrate the level of microbiome-associated metabolites in relation to scores in executive functions (A), ADHD symptoms (B), and social behavior (C) as well as heavy metal load (D) for each individual child. Each dot represents scores of a child who took part in both measures. Outlier values of the boy excluded from correlation and path analyses are marked by a red dot. In each chart, children with a higher level of microbiome-associated metabolites are represented by dots that are higher up. Children with worse executive functions (A), more ADHD symptoms (B), or higher heavy metal load (D) are represented by dots to the right; children with more unsocial behavior (C), to the left.

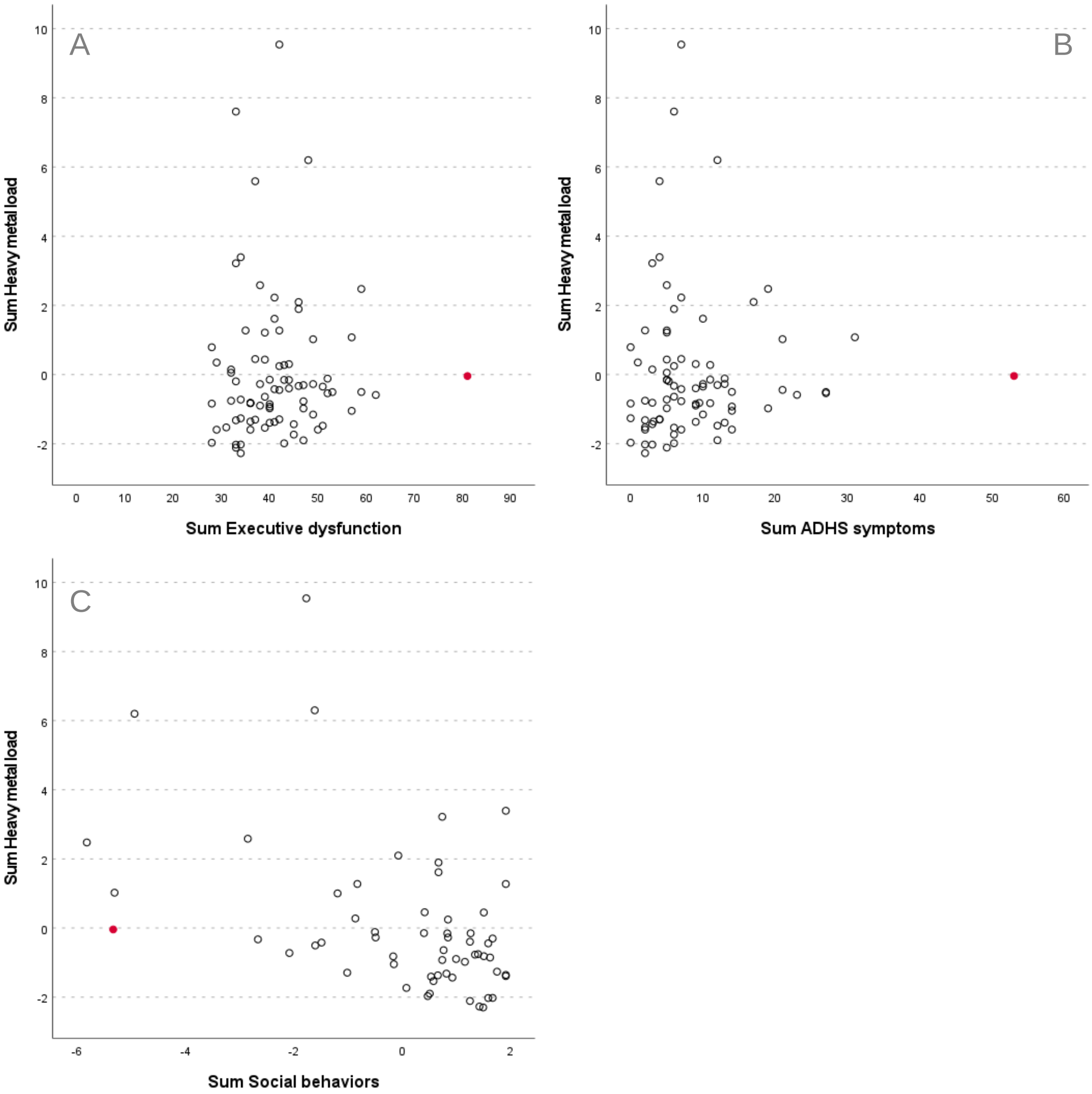

Supplementary Figure 3. Relationships between heavy metal load and behavioral scores. The scatter plots illustrate the level of heavy metal load in relation to scores in ADHD symptoms (A), social behavior (B), and executive functions (C) for each individual child. Each dot represents scores of a child who took part in both measures. Outlier values of the boy excluded from correlation and path analyses are marked by a red dot. In each chart, children with higher heavy metal load are represented by dots that are higher up. Children with worse executive functions (A) or more ADHD symptoms (B) are represented by dots to the right; children with more unsocial behavior (C), to the left.

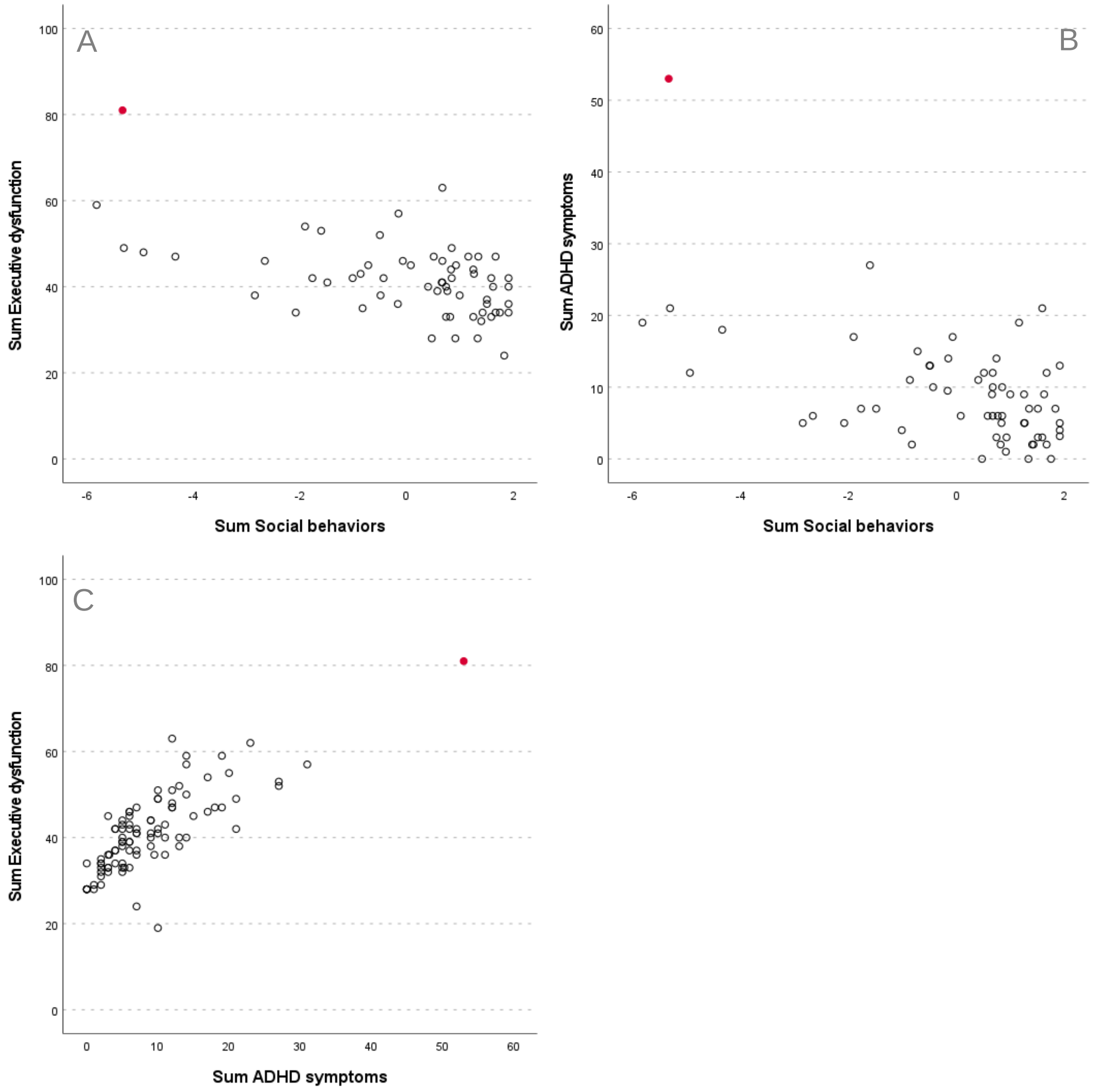

Supplementary Figure 4. Interrelationships within behavioral scores. The scatter plots illustrate the moderate relationships between social behaviors and scores in executive functions (A) and ADHD symptoms (B), as well as the highly correlated ADHD symptoms and executive dysfunction scores (C; $r = .77$, $p < .001$) for each individual child. Each dot represents scores of a child who took part in both measures. Outlier values of the boy excluded from correlation and path analyses are marked by a red dot. In the charts, children with worse executive functions (A, C) or more ADHD symptoms (B) are represented by dots that are higher up. In chart C children with more ADHD symptoms are represented by dots to the right; in charts A and B children with more unsocial behaviour, to the left.
